# Evaluation of Factors Associated With Short-term Failure After Primary Isolated PCL Reconstruction: A Study of Patients From the Swedish and Norwegian Knee Ligament Registries

**DOI:** 10.1177/23259671241305191

**Published:** 2025-01-03

**Authors:** Bálint Zsidai, Philipp W. Winkler, Eric Naarup, Ebba Olsson, Alexandra Horvath, Gilbert Moatshe, Martin Lind, Volker Musahl, Eric Hamrin Senorski, Kristian Samuelsson

**Affiliations:** † Department of Orthopaedics, Institute of Clinical Sciences, Sahlgrenska Academy, University of Gothenburg, Gothenburg, Sweden; ‡ Sahlgrenska Sports Medicine Center, Gothenburg, Sweden; § Department for Orthopaedics and Traumatology, Kepler University Hospital GmbH, Johannes Kepler University Linz, Linz, Austria; ‖ Department of Orthopaedics, Sahlgrenska University Hospital, Mölndal, Sweden; ¶ Oslo University Hospital and University of Oslo, Oslo, Norway; # Oslo Sports Trauma Research Center, Norwegian School of Sports Sciences, Oslo, Norway; ** Department of Orthopaedic Surgery, Aarhus University Hospital, Aarhus N, Denmark; †† Department of Orthopaedic Surgery, UPMC Freddie Fu Sports Medicine Center, University of Pittsburgh, Pittsburgh, USA; ‡‡ Unit of Physiotherapy, Department of Health and Rehabilitation, Institute of Neuroscience and Physiology, Sahlgrenska Academy, University of Gothenburg, Gothenburg, Sweden; §§ Sportrehab Sports Medicine Clinic, Gothenburg, Sweden; Investigation performed at Institute of Clinical Sciences, Sahlgrenska Academy, University of Gothenburg, Gothenburg, Sweden

**Keywords:** posterior cruciate ligament, knee ligament reconstruction, patient-reported outcomes, surgical failure, clinical failure, injury mechanism

## Abstract

**Background:**

The rate of subjective failure after isolated primary posterior cruciate ligament reconstruction (PCL-R) is relatively high, requiring an improved understanding of factors associated with inferior outcomes.

**Purpose:**

To determine the association between patient and injury-related factors and total (surgical and clinical) failure at 2 years after PCL-R based on data from the Swedish National Knee Ligament Registry (SNKLR) and the Norwegian Knee Ligament Registry (NKLR).

**Study Design:**

Cohort study; Level of evidence, 3.

**Methods:**

Patients with primary isolated PCL-R registered between January 1, 2004 (NKLR), or January 1, 2005 (SNKLR), and December 31, 2020, were included. The primary study outcome was the risk of PCL-R failure at the 2-year follow-up, either surgical (≤2 years of index surgery) or clinical (Knee injury and Osteoarthritis Outcome Score [KOOS] Quality of Life subscale [QoL] <44) failure. Risk factors for failure were estimated utilizing univariable and multivariable logistic regression analyses.

**Results:**

Among the 189 included patients (36.0% from the SNKLR and 64.0% from the NKLR), the rate of 2-year surgical failure was 5.8%, while the rate of clinical failure was 45.0%. Multivariable analysis showed a negative association between the baseline KOOS QoL and the risk of PCL-R failure (OR, 0.74; 95% CI, 0.57-0.97; *P* = .027). Univariable analysis indicated a positive association between traffic-related injury mechanism and PCL-R failure risk (OR, 3.11; 95% CI, 1.48-6.50; *P* = .0026), with a further positive association shown in the adjusted (OR, 6.08; 95% CI, 2.00-18.50; *P* = .0015) and multivariable (OR, 6.11; 95% CI, 2.01-18.55; *P* = .0014) models. An area under the curve of 0.70 (95% CI, 0.60-0.80) was reported for the final multivariable model, implying at best poor to acceptable ability of the model to estimate PCL-R failure risk based on the variables considered.

**Conclusion:**

Patients with isolated primary PCL-R had a high (45%) rate of short-term clinical failure, and traffic-related injury was associated with increased odds of failure. No modifiable risk factors were determined as potential predictors of failure. Clinicians treating patients with isolated PCL-R associated with a traffic-related injury mechanism should be aware of a >6-fold increased odds of revision surgery and inferior knee-related quality of life at short-term follow-up.

Isolated posterior cruciate ligament (PCL) reconstruction (PCL-R) accounts for one-third to approximately one-half of all surgically reconstructed PCL tears,^13,17,24^ with considerable heterogeneity in terms of patient characteristics, injury-related factors, and associated injuries at presentation.^13,17,32^ Previous reports determined important surgical risk factors for PCL-R failure, such as posterolateral corner deficiency, suboptimal graft tunnel position, concomitant varus malalignment, and low posterior tibial slope.^16,28^ Additionally, the majority of patients are exposed to complicating factors associated with revision surgery, including impaired activities of daily living, prior meniscectomy, arthritis, and associated ligamentous deficiencies.^16^ However, the associations between modifiable and nonmodifiable patient and injury-related factors and surgical and clinical failure after PCL-R are scarcely investigated. Historically, a traffic-related injury mechanism was attributed a major role in the incidence of PCL tears.^5,25^ However, recent registry studies have underscored the important role of sports-related PCL injury mechanisms.^13,17,32^

While a comparable rate of postoperative improvement in subjective knee function was reported among patients with PCL-R and anterior cruciate ligament reconstruction (ACL-R),^29^ patients with PCL-R report inferior preoperative and postoperative subjective knee function.^18^ Consequently, patients with PCL tears may experience suboptimal recovery of knee function after surgical treatment despite a rate of improvement similar to that of patients with ACL-R.^18^ Despite a considerable risk of short-term surgical failure and a high rate of clinical failure due to impaired knee function in the primary isolated PCL-R population,^13,14^ risk factors for surgical and clinical treatment failure are underreported.

Diverse injury mechanisms, patient variables, perceived preoperative knee function, and variability in concomitant ligamentous and intra-articular injury patterns in patients with PCL-R^12,13,32^ suggest that complex, synergistic relationships may exist among these impactful variables. For instance, 1 recent study in patients with multiligament PCL-R found that delayed time from injury to surgery was associated with a greater prevalence of chondral lesions reported intraoperatively.^31^ The high variability in the prevalence and management of concomitant ligamentous injuries in the setting of multiligament PCL-R may lead to considerable confounding, and risk factors for inferior patient outcomes may be easier to interpret in the setting of isolated PCL-R.

The objective of this study was to determine the association between patient and injury-related factors and total (surgical and clinical) failure at the 2-year follow-up after primary isolated PCL-R utilizing data from the Scandinavian knee ligament registries.

## Methods

Ethics approval for the study was granted by the Swedish and Norwegian ethical review authorities. The study was performed in accordance with the Declaration of Helsinki. Results of the performed analyses were reported in accordance with the STROBE (Strengthening the Reporting of Observational Studies in Epidemiology) checklist.^27^ Patients registered in the Swedish National Knee Ligament Registry (SNKLR) and the Norwegian Knee Ligament Registry (NKLR) between January 1, 2004 (NKLR), or January 1, 2005 (SNKLR), and December 31, 2020, were included. Patients were excluded from the study cohort if they registered for interventions other than primary isolated PCL-R, were missing preoperative baseline data for the Knee injury and Osteoarthritis Outcome Score (KOOS) Quality of Life (QoL) subscale, underwent PCL-R in combination with other ligament injuries without known management, underwent revision PCL-R, or had concomitant fractures of the femur, patella, fibula, or tibia. Furthermore, patients with missing 2-year KOOS QoL were excluded, unless a new PCL-R was registered for the same patient within the 2-year interval. The final study cohort was further stratified into 2 subgroups to compare patients without PCL-R (surgical or clinical) failure at the time of the 2-year follow-up and those with PCL-R failure (surgical or clinical) within 2 years of the index surgery.

### Scandinavian Knee Ligament Reconstruction Registries

The NKLR was established in 2004, followed by the SNKLR in 2005, to collect prospective data on patients undergoing reconstructive knee ligament surgery in Scandinavia, primarily patients with ACL-R. The aim of these registries was to provide a platform for the assessment of patient outcomes after surgical interventions with respect to patient, injury-related, and surgical variables. Through determining factors with potential impact on prospectively reported functional outcomes, surgeons performing knee ligament reconstructions can synthesize information to guide patient management. Data collection is a 2-part process across the 2 registries. By completion of a mail- or web-based survey, patients contribute information related to baseline characteristics, injury-related activity, and preoperative and postoperative knee function at intervals of 1, 2, 5, and 10 years after the most recently performed surgery. Additionally, surgeons report data related to the surgical interventions and specific surgical techniques performed, as well as the prevalence of concomitant injuries and their treatment. Both the Swedish and Norwegian national registries are described in further detail elsewhere.^1,7,8^ In accordance with Norwegian legislation, participation in the NKLR requires the completion of a conflict of interest form. While informed consent is not a prerequisite of data collection in the SNKLR, patients may request exemption from the registries in writing.

### Study Variables

The following patient variables were considered for between-group comparison and risk factor selection in the final model: patient age at the time of surgery, sex, body mass index (BMI), injury laterality, time from injury to surgery (months), injury mechanism (Table 1), and preoperative KOOS. The KOOS is a patient-reported outcome measure used to assess subjective knee function in patients with knee-related conditions and interventions.^22^ The 5 subscales of the KOOS used for reporting subjective knee function are Pain, Symptoms, ADL, Sports/Recreation, and QoL. Each subscale is graded between 0 and 100 points, with the former representing the worst possible state and the latter the complete absence of impaired subjective knee function. While clinically acceptable psychometric properties have been reported for the KOOS in patients with knee injuries,^21^ evidence is limited regarding the specific use of the KOOS for patients with PCL injuries in terms of content validity, appropriateness, and interpretability. The test-retest reliability of the KOOS was determined to be high, with intraclass correlation coefficients between 0.61 and 0.95 across subscales.^3^ Additionally, responsiveness assessed based on minimal detectable change was reported to range from 14.3 to 19.6 for younger patients and ≥20 for older patients.^3^

**Table 1:** Injury Mechanism Categories Based on Activities Performed at the Time of PCL Injury^*a*^

Injury Mechanism Category	Registered Activity
Alpine/skiing	Alpine skiing, telemark, twin tip
Pivoting sport	American football, basketball, bandy, dancing, football, floorball, handball, ice hockey, martial arts, racket sports, volleyball, wrestling
Nonpivoting sport	Cross-country skiing, cycling, enduro/motorcross, equestrian/horseback riding, skateboarding, snowboarding, wakeboarding
Other physical activity	Other free-time/physical activity, exercise, trampoline, other team sport
Other	Other, outdoor activity, work-related, fall, jumping, play
Traffic	Traffic-related injury

a Categories are described as in the Swedish National Knee Ligament Registry and the Norwegian Knee Ligament Registry. PCL, posterior cruciate ligament.

### Outcome Measures

The primary outcome of the study was the prevalence of total isolated PCL-R failure at the 2-year follow-up, defined either as surgical failure (new PCL-R registered for the same patient within 2 years of index surgery) or clinical failure (KOOS QoL <44). A KOOS QoL <44 was previously used to define clinical failure in studies assessing postoperative subjective knee function in the setting of PCL-R^13,14^; thus, this value was consequently chosen as a proxy for clinical failure after primary isolated PCL-R in the present study.

### Statistical Analysis

Data analysis was performed with SAS/STAT (Version 9.4; SAS Institute Inc). Figures were created with the R statistical computing software^20^ and the RStudio software package.^23^ Deidentified data were separately queried from the SNKLR and NKLR, with subsequent merger of the separate data sets performed by the lead statistician of the project. Categorical variables are reported as frequencies with percentages, and continuous variables are reported as means with standard deviations or medians with ranges, as appropriate for the given variable. For comparisons between groups, the Fisher exact test (lowest 1-sided *P* value multiplied by 2) was used for dichotomous variables, and the Fisher nonparametric permutation test was used for continuous variables. The confidence intervals for change in between-group proportions consisted of the unconditional exact confidence limits. When no exact limits were possible to compute, the asymptotic Wald confidence limits with continuity correction were calculated instead. Confidence intervals for mean between-group differences were based on the Fisher nonparametric permutation test.

The impacts of the analyzed variables on total failure risk at the 2-year follow-up were estimated with univariable and stepwise multivariable logistic regression models and reported as odds ratios with 95% confidence intervals. Adjusted univariable analysis was performed with regard to potential confounders selected a priori, such as source registry, sex, and baseline KOOS QoL, using logistic regression. The Firth penalized maximum likelihood estimation was performed to handle events for which an insufficient number of data points were available for odds ratios estimation (quasi-complete separation of data points).^6^ All tests were 2-sided and conducted at the 5% significance level. *P* values, odds ratios, and area under the receiver operating characteristic curve (AUC) measurements were based on original values and not on stratified groups, where odds ratio was defined as the ratio for the odds of a 10-unit increase in the assessed risk factor. Values reported for the AUC were interpreted as 0.5 (no discrimination, ie, equivalent to random chance), >0.5 to <0.7 (poor discrimination), ≥0.7 to <0.8 (acceptable discrimination), ≥0.8 to <0.9 (excellent discrimination), ≥0.9 to <1.0 (outstanding discrimination), and 1.0 (perfect discrimination).^9^

## Results

**Figure 1.**
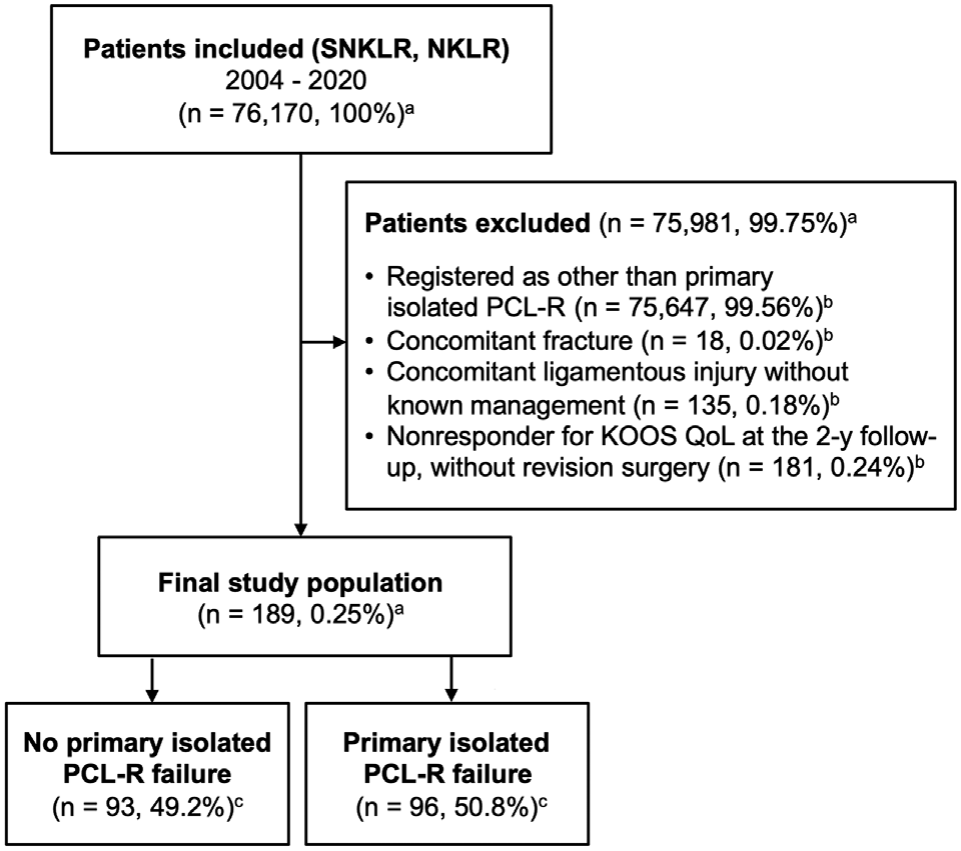
Flowchart of patients included and excluded from the study population. Values are presented as count and proportion. ^a^Percentage of patients included. ^b^Percentage of total fulfilled exclusion criteria. ^c^Percentage of patients considered in the final study population. KOOS, Knee injury and Osteoarthritis Outcome Score; NKLR, Norwegian Knee Ligament Reconstruction Registry; PCL-R, posterior cruciate ligament reconstruction; SNKLR, Swedish National Knee Ligament Registry.

A total of 76,170 patients were included from the SNKLR and NKLR. After the application of exclusion criteria, the final analysis included 189 patients with primary isolated PCL-R, 68 (36.0%) from the SNKLR and 121 (64.0%) from the NKLR. Stratification of the study cohort based on the prevalence of 2-year isolated PCL-R failure resulted in 93 (49.2%) patients without and 96 (50.8%) patients with PCL-R failure (Figure 1). Relative to the total study population, the rate of 2-year surgical failure was 5.8% (n = 11), while the rate of clinical failure was 45.0% (n = 85). In the patient subgroup with PCL-R failure, the proportional rates of surgical and clinical PCL-R were 11.5% (n = 11) and 88.5% (n = 85), respectively (Table 2).

**Table 2:** Patient Variables, Injury-Related Variables, Baseline Subjective Knee Function, and the Prevalence of Clinical and Surgical PCL-R Failure at the 2-Year Follow-up After Primary Isolated PCL-R^*a*^

Variable	No PCL-R Failure (n = 93)	PCL-R Failure (n = 96)	*P*
Age at surgery, y	28.6 ± 12.0	31.2 ± 12.1	.14
Sex			
Male	48 (51.6)	49 (51.0)	
Female	45 (48.4)	47 (49.0)	≥.999
BMI, kg/m^2^	25.6 ± 5.2; n = 71	25.5 ± 4.8; n = 68	.90
KOOS at baseline			
Symptoms	68.0 ± 17.5; n = 62	62.8 ± 17.8; n = 60	.12
Pain	63.6 ± 17.3; n = 62	56.4 ± 16.9; n = 60	.024
Sports/Rec	30.8 ± 25.1; n = 62	23.8 ± 20.2; n = 59	.091
QoL	32.0 ± 16.0; n = 62	25.8 ± 14.9; n = 59	.031
ADL	73.9 ± 17.4; n = 62	68.2 ± 18.4; n = 60	.077
KOOS QoL at 2-y follow-up	71.6 ± 13.1; n = 93	29.9 ± 12.7; n = 85	—
Injury side			
Right	46 (49.5)	49 (51.0)	
Left	47 (50.5)	47 (49.0)	.94
Time to surgery, mo	15.2 [0.3-241.1]; n = 92	21.8 [0.5-307.2]; n = 91	.46
Injury mechanism^*b*^			
Alpine/skiing	7 (7.5)	8 (8.3)	≥.999
Pivoting sport	34 (36.6)	27 (28.1)	.23
Nonpivoting sport	8 (8.6)	3 (3.1)	.18
Other physical activity	13 (14.0)	9 (9.4)	.42
Other	16 (17.2)	19 (19.8)	.84
Traffic	13 (14.0)	30 (31.3)	.0092
Missing	2 (2.2)	0 (0)	—
Total failure	—	96 (100)	—
Surgical failure	—	11 (11.5)	—
Clinical failure	—	85 (88.5)	—

a Data are presented as mean ± SD, n (%), or median [range]. Sample sizes are given when they differ from that of the study group. ADL, Activities of Daily Living; BMI, body mass index; KOOS, Knee injury and Osteoarthritis Outcome Score; PCL-R, posterior cruciate ligament reconstruction; QoL, Quality of Life; Rec, Recreation. Dashes indicate that the *p*-values are not calculated between the two groups, since the groups are defined based on cutoff values for the same variable.

b See Table 1 for activities related to these injury mechanism categories.

### Patient Characteristics and Baseline Subjective Knee Function

**Figure 2.**
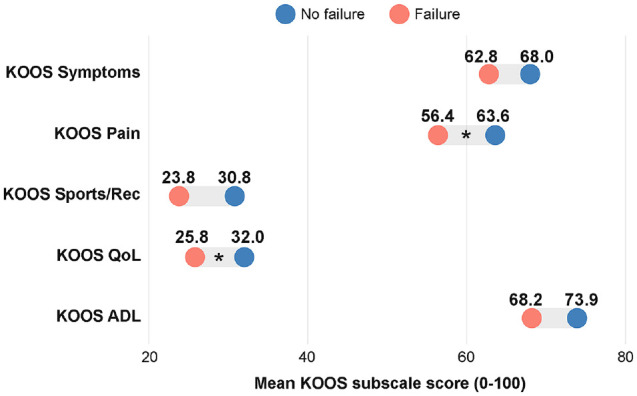
Dumbbell chart for comparison of mean preoperative values reported on the Knee injury and Osteoarthritis Outcome Score (KOOS) subscales (0-100) between patients without and with total primary isolated posterior cruciate ligament reconstruction failure (surgical or clinical) at the 2-year follow-up. *Statistically significant between-group difference. ADL, Activities of Daily Living; QoL, Quality of Life; Rec, Recreation.

The mean age of patients with and without PCL-R failure was 31.2 ± 12.1 years and 28.6 ± 12.0 years, respectively (*P* = .14). There were no statistically significant between-group differences in patient factors such as age, patient sex, BMI, injury laterality, or time from injury to surgery (Table 2). Values for the KOOS Symptoms, Sports/Recreation, and ADL subscales were comparable between patient groups (Table 2). A statistically significant difference in the KOOS Pain subscale was seen between patients with PCL-R failure (56.4 ± 16.9) and those without (63.6 ± 17.3) (*P* = .024). At baseline, there was a statistically significant between-group difference in the KOOS QoL subscale: 32.0 ± 16.0 for patients without and 25.8 ± 14.9 for patients with PCL-R failure (*P* = .031) (Figure 2). At the 2-year follow-up, the KOOS QoL was 71.6 ± 13.1 for patients without PCL-R failure and 29.9 ± 12.7 for patients in the PCL-R failure group (Table 2).

### Injury Mechanism

**Figure 3.**
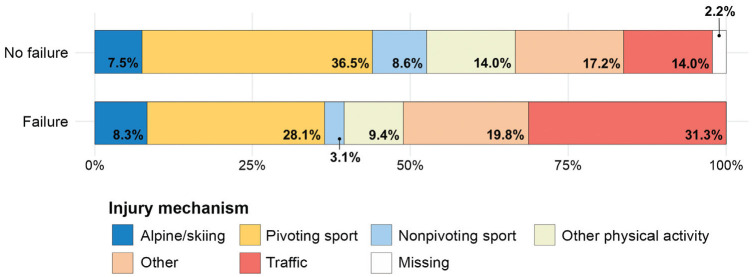
Stacked bar chart of injury mechanism prevalence in patients without and with total primary isolated posterior cruciate ligament reconstruction failure (surgical or clinical) at the 2-year follow-up.

Between-group comparisons of the distribution of injury mechanisms in patients with isolated primary PCL-R are presented in Table 2. The prevalence of alpine/skiing, pivoting, nonpivoting, other physical activities, and other injury mechanisms was comparable between patients with and without 2-year PCL-R failure (Table 2). Traffic-related injury showed a significantly greater prevalence in the patients with 2-year PCL-R failure, with 30 (31.3%) cases compared with 13 (14.0%) cases in patients without PCL-R failure (*P* = .0092). There were 2 (2.2%) patients with missing data with regard to injury mechanism in the patient group without PCL-R failure (Figure 3).

### Risk Factor Assessment of PCL-R Failure

Univariable, adjusted, and multivariable logistic regression analyses showed no significant association between patient age, patient sex, BMI, injury laterality, and time from injury to surgery with the risk of 2-year PCL-R failure (Tables 3 and 4). Furthermore, univariable, adjusted, and multivariable logistic regression did not a show relationship of alpine/skiing, pivoting sport, nonpivoting sport, other physical activity, or other injury mechanisms with PCL-R failure risk. Univariable (OR, 3.11 [95% CI, 1.48-6.50]; *P* = .0026), adjusted (OR, 6.08 [95% CI, 2.00-18.50]; *P* = .0015), and multivariable (OR, 6.11 [95% CI, 2.01-18.55]; *P* = .0014) analyses showed a positive association between traffic-related injury and the risk of 2-year PCL-R failure. When considering baseline preoperative KOOS subscales, the Symptoms, Sports/Recreation, and ADL subscales did not show an association with PCL-R failure risk based on univariable, adjusted, and multivariable logistic regression models. While univariable logistic regression showed a negative association between the KOOS Pain (OR, 0.78 [95% CI, 0.63-0.98]; *P* = .029) and QoL (OR, 0.78 [95% CI, 0.95-1.00]; *P* = .049) subscales, these relationships were lost with the adjusted logistic regression model. Multivariable analysis showed a negative association between the KOOS QoL subscale and PCL-R failure (OR, 0.74 [95% CI, 0.57-0.97]; *P* = .027). An AUC of 0.70 (95% CI, 0.60-0.80) was reported for the final multivariable logistic regression model.

**Table 3:** Results of Univariable Logistic Regression Models to Determine the Impact of Patient Variables, Injury-Related Variables, and Baseline Subjective Knee Function on the Prevalence of Primary Isolated PCL-R Failure at the 2-Year Follow-up^*a*^

Risk Factor	PCL-R Failure, n (%)	Univariable^*b*^		Adjusted^*c*^		
		OR (95% CI)	*P*	OR (95% CI)	*P*	AUC (95% CI)
Age at surgery, y		1.15 (0.90-1.46)	.27	1.04 (0.73-1.48)	.82	0.56 (0.48-0.65)
14.0 to <21.0	25 (41.7)					
21.0 to <34.0	31 (52.5)					
34.0 to 60.0	29 (49.2)					
Sex		1.14 (0.64-2.06)	.65	1.39 (0.66-2.94)	.39	0.52 (0.44-0.59)
Male	41 (46.1)					
Female	44 (49.4)					
BMI, kg/m^2^		0.99 (0.93-1.06)	.83	0.92 (0.82-1.03)	.15	0.51 (0.41-0.61)
16.2 to <19.5	2 (100)					
19.5 to <25.0	32 (45.1)					
25.0 to <30.1	15 (41.7)					
30.1 to 53.7	12 (52.2)					
Injured side		1.05 (0.58-1.89)	.87	1.14 (0.53-2.42)	.74	0.51 (0.43-0.58)
Right	41 (47.1)					
Left	44 (48.4)					
Time to surgery, mo		1.00 (1.00-1.01)	.38	1.00 (0.99-1.01)	.49	0.59 (0.50-0.68)
0.3 to <12.1	16 (32.0)					
12.1 to <24.2	25 (49.0)					
24.2 to 307.2	39 (54.9)					
Injury mechanism						
Alpine/skiing	8 (53.3)	1.25 (0.43-3.60)	.68	0.78 (0.22-2.79)	.70	0.51 (0.47-0.55)
Pivoting sport	25 (42.4)	0.70 (0.37-1.31)	.27	0.62 (0.28-1.37)	.24	0.54 (0.47-0.61)
Nonpivoting sport	1 (11.1)	0.12 (0.02-1.01)	.051	0.09 (0.00-2.25)^*d*^	.14	0.54 (0.51-0.57)
Other physical activity	5 (27.8)	0.38 (0.13-1.10)	.074	0.40 (0.12-1.38)	.15	0.54 (0.50-0.59)
Other	17 (51.5)	1.17 (0.55-2.50)	.68	1.43 (0.51-4.06)	.50	0.51 (0.45-0.57)
Traffic	29 (69.0)	3.11 (1.48-6.50)	.0026	6.08 (2.00-18.50)	.0015	0.60 (0.54-0.66)
Baseline KOOS Symptoms		0.84 (0.68-1.04)	.11	0.94 (0.74-1.19)	.62	0.58 (0.47-0.68)
7.1 to <60.7	20 (55.6)					
60.7 to <75.0	19 (46.3)					
75.0 to 100.0	16 (40.0)					
Baseline KOOS Pain		0.78 (0.63-0.98)	.029	0.85 (0.64-1.13)	.26	0.61 (0.51-0.71)
8.3 to <53.1	24 (58.5)					
53.1 to <66.7	17 (41.5)					
66.7 to 100.0	14 (40.0)					
Baseline KOOS Sports/Rec		0.87 (0.74-1.03)	.11	1.00 (0.98-1.02)	.87	0.58 (0.47-0.68)
0.0 to <15.0	20 (54.1)					
15.0 to <35.0	16 (43.2)					
35.0 to 100.0	18 (42.9)					
Baseline KOOS ADL		0.83 (0.68-1.02)	.082	0.92 (0.71-1.19)	.51	0.58 (0.47-0.68)
20.6 to <64.7	20 (52.6)					
64.7 to <82.4	20 (48.8)					
82.4 to 100.0	15 (39.5)					
Baseline KOOS QoL		0.78 (0.95-1.00)	.049	0.79 (0.95-1.00)	.060	0.60 (0.50-0.71)
<25.0	23 (59.0)					
25.0 to <37.5	14 (41.2)					
37.5 to 75.0	17 (39.5)					

a OR, area under the receiver operating characteristic curve (AUC), and *P* values are based on original values and not on stratified groups. The odds ratio is the ratio for the odds of a 10-unit increase in the assessed risk facor. ADL, Activities of Daily Living; BMI, body mass index; KOOS, Knee injury and Osteoarthritis Outcome Score; PCL-R, posterior cruciate ligament reconstruction; QoL, Quality of Life; Rec, Recreation.

b All tests were performed with univariable logistic regression.

c Adjusted for source register, sex, and baseline KOOS QoL using logistic regression.

d Values estimated using the Firth penalized maximum likelihood estimation due to quasi-complete separation of data points.

**Table 4:** Results of Multivariable Logistic Regression Model for Traffic-Related Injury and Baseline KOOS QoL^*a*^

Risk Factor	OR (95% CI)	*P*
Traffic	6.11 (2.01-18.55)	.0014
KOOS QoL	0.74 (0.57-0.97)	.027

a AUC for multivariable model = 0.70 (95% CI, 0.60-0.80). KOOS, Knee injury and Osteoarthritis Outcome Score; QoL, Quality of Life.

## Discussion

The main finding of the study was that the odds of 2-year surgical or clinical primary isolated PCL-R failure were >6-fold greater in patients with traffic-related injury mechanisms compared with those with other types of injury mechanism. While significant between-group differences between patients without and with 2-year isolated primary PCL-R failure were observed in terms of baseline KOOS Pain and QoL subscales, and traffic-related injury prevalence, the final stepwise multivariable model only included the preoperative KOOS QoL subscale and traffic-related injury mechanism as potential risk factors. The AUC of 0.70 for the presented multivariable regression model implies, at best, poor to acceptable ability of the multivariable model to discriminate total PCL-R failure risk based on the included variables.^9^ No modifiable or nonmodifiable patient risk factors of PCL-R failure were identified. However, the results of the current study underscore the important role of a traffic-related injury mechanism as clinically relevant, and a predisposing factor for inferior short-term subjective knee function and early revision surgery after primary isolated PCL-R. Furthermore, there was a relatively high rate of clinical failure (45%) in patients with primary isolated PCL-R at the 2-year follow-up.

A limited volume of data from the existing literature estimates the rate of short- to midterm surgical failure after primary isolated PCL-R to be as frequent as 3% to 12.6%.^13,26,30^ While an overall 2-year surgical failure rate of 5.8% was found in the current study, the total failure rate was considerably magnified to 50.8% when a proxy for clinical failure, defined as KOOS QoL <44 was introduced. This finding is in alignment with 1 recent study from the NKLR and DKLR, which found a 2-year subjective failure rate of 49.5% in terms of KOOS QoL <44, after isolated primary PCL-R.^14^ Another study from the DKLR estimated the 5-year rate of subjective failure (KOOS QoL <44) after primary isolated PCL-R to be somewhat reduced, at 35%, with a 5-year revision rate of 3%.^13^ Furthermore, the rate of overall isolated PCL-R failure in a military population with a mean follow-up of 19.5 months was 42.7%, when considering both revision surgery and medical discharge due to impairment in knee function.^26^ These findings are comparable to the high rate of clinical failure reported in the present study. While it is not possible to infer causal relationships for the high rate of surgical and clinical failure determined by the current and previous studies, it is plausible that they depend on the synergistic interaction of multiple predisposing factors and postoperative exposures, many of which are not captured in the Scandinavian registries. A complex interaction between modifiable and nonmodifiable anatomic^2,10,28^ and patient characteristics,^13,32^ injury mechanism,^24^ concomitant injuries,^32^ objectively measured posterior knee laxity,^11^ and surgical techniques^4,30^ is likely at play. The impacts of these measurable factors are presumably further modified by the influences of variables that are difficult to quantify, such as surgeon experience; the quality, intensity, and timeliness of postoperative rehabilitation and return to preinjury level of activity; and patient compliance.^19,30^ Nonetheless, multisite registry data and statistical methods may provide helpful information for the detection of patient subgroups at an increased risk of failure, and predictors associated with inferior outcomes.

The total prevalence of a traffic-related injury mechanism was 23% in the present study, with a proportionally greater prevalence of traffic-related injuries in the PCL-R failure patient group (31.3%) compared with the group without surgical or clinical failure (14.0%). The current literature estimates the rate of traffic-related injury mechanisms in the setting of primary isolated and multiligament PCL-R to be in the range of approximately 20% to 35%.^13,24,32^ However, the considerably lower proportion of traffic-related injuries in the setting of isolated PCL-Rs in the current analysis is in alignment with the notion that the majority of surgically treated PCL injuries where other ligaments are not involved are caused by sports-related injury mechanisms.

Previous reports from the Scandinavian knee ligament reconstruction registries found significant short-term improvements in subjective knee function after isolated primary PCL-R. One recent study from the SNKLR reported pronounced improvements across the KOOS Sports/Recreation (mean, +20 points) and QoL (mean, +23 points) subscales at the 2-year follow-up.^29^ Despite these findings, the majority of patients with 2-year PCL-R failure (88.5%) reported clinically inadequate knee function, with insufficient improvement in knee-related quality of life (KOOS QoL <44 points). Previous registry studies have drawn attention to the relatively high prevalence of meniscal and chondral injuries in the setting of surgically treated isolated and multiligament PCL tears.^31,32^ Consequently, intra-articular injury is likely to impact the short-term postoperative knee-related quality of life in patients with primary isolated PCL-R, especially in the setting of high-energy, traffic-related injury mechanisms that may lead to concurrent meniscal and chondral lesions.^32^

### Strengths and Limitations

The strengths of the presented study include strictly applied inclusion and exclusion criteria and a cross-sectional study design to determine the impact of demographic and injury-related variables on short-term PCL-R failure in an international study cohort. Additionally, the current analysis benefits from a greater sample size and reduced patient heterogeneity in terms of PCL tear profile^12^ compared with previous studies,^15,24^ which is essential for determining clinically relevant predictors for patients with isolated primary PCL-R.

While the study design ensures adjustment for confounding demographic and injury-related variables registered in the SNKLR and NKLR, consideration of additional surgical confounders that may affect postoperative knee function—and thereby 2-year clinical PCL-R failure per definition of the study—exceeded the scope of the study design. Therefore, future studies are required to assess the independent and synergistic roles of surgical variables and concurrent intra-articular pathology on surgical and clinical PCL-R failure. Furthermore, the current study suffers from the incomplete coverage of all patients with PCL-R, as 181 patients (Figure 1) were lost to follow-up due to unreported 2-year subjective knee function (specifically the KOOS QoL subscale) in the registries. This aspect of the study amounts to a 49% exclusion of otherwise eligible patients for the study and increases the risk of selection bias due to patient attrition. Importantly, the prevalence of total PCL-R failure was 50.8%, with the majority defined as clinical failure. This high rate of clinical failure strongly suggests that patients with sufficient short-term clinical improvement are lost to follow-up, ultimately resulting in overestimation of the total isolated primary PCL-R failure rate. Therefore, further studies based on complete, prospectively collected data with measures taken to eliminate the uncertainty caused by patient attrition are required to reliably assess the primary isolated PCL-R failure rate and pinpoint risk factors for surgical and clinical failure. Finally, the analyzed data did not contain information about preoperative objective anteroposterior laxity measurements, data from stress radiographs, and clear indications for isolated primary PCL-R, other than the mechanism of injury. Future analyses would benefit from the inclusion of such variables for the clarification of clinically relevant predictors.

## Conclusion

Patients with isolated primary PCL-R were found to have a high (45%) rate of short-term clinical failure, and traffic-related injury was associated with increased odds of PCL-R failure at 2 years postoperatively. Clinicians treating patients with isolated PCL-R associated with a traffic-related injury mechanism should be aware of a >6-fold increased odds of revision surgery and inferior knee-related quality of life at short-term follow-up and counsel patients accordingly.
